# Two Endotracheal Tubes in One Trachea with a Traumatic Injury

**DOI:** 10.1155/2021/9912553

**Published:** 2021-05-11

**Authors:** Andrew Winegarner, Harish Lecamwasam, Mark C. Kendall, Shyamal Asher

**Affiliations:** Department of Anesthesiology, Rhode Island Hospital, Warren Alpert School of Medicine at Brown University, Providence, RI, USA

## Abstract

**Background:**

Traumatic airway injuries often require improvising solutions to altered anatomy under strict time constraints. We describe here the use of two endotracheal tubes simultaneously in the trachea to facilitate securing an airway which has been severely compromised by a self-inflicted wound to the trachea. *Case Presentation*: A 71-year-old male presented with a self-inflicted incision to his neck, cutting deep into the trachea itself. An endotracheal tube was emergently placed through the self-inflicted hole in the trachea in the ED. The patient was bleeding profusely, severely somnolent, and desaturating upon arrival to the operating room. Preservation of the tenuous airway was a priority while seeking to establish a more secure one. A video laryngoscope was used to gain a wide view of the posterior oropharynx and assist with oral intubation using a fiberoptic scope loaded with a second endotracheal tube. The initial tube's cuff was deflated as the second tube was advanced over the fiberoptic scope, thereby securing the airway while a completion tracheostomy was performed.

**Conclusions:**

Direct penetrating airway trauma may necessitate early, albeit less secure, intubations though the neck wounds prior to operating room arrival. The conundrum is weighing the risk of losing a temporary airway while attempting to establish a more secure airway. Here, we demonstrate the versatility of common anesthesia tools such as a video laryngoscope and a fiberoptic bronchoscope and the welcome discovery of the trachea's ability to accommodate two endotracheal tubes simultaneously so as to ensure a patent airway at all points throughout resuscitation.

## 1. Introduction

Traumatic airways can be an especially formidable challenge to anesthesiologists. These injuries can require emergent interventions in the field or Emergency Department (ED) to ensure adequate oxygenation and ventilation. Emergent attempts to secure an airway can complicate a more long-term solution or prevent a comprehensive evaluation of airway injury. When such a patient presents to the operating room (OR), the anesthesiologist must decide how to handle the emergently placed airway and, if needed, be able to replace it with a more secure and reliable airway. We present a case of a patient with a self-inflicted, traumatic tracheal injury where an endotracheal tube (ETT) was placed emergently in the ED through the injury site. We describe the simultaneous use of a video laryngoscope (VL) and fiberoptic bronchoscope (FOB) to evaluate the extent of the tracheal injury and the placement of two ETTs in the trachea to allow for safe transition from the emergently placed airway to a more secure one.

## 2. Case Presentation

A 71-year-old 5'6, 80 kg male with an unknown medical history presented to the ED with a self-inflicted stab wound to the neck resulting in a 3 cm laceration across the trachea. In the ED, the patient had an 8.0 ETT placed into the trachea through the tracheal injury and was emergently brought to the OR. The extent of the tracheal injury and any associated damage to adjacent structures was unknown at the time. In the OR, the existing ETT could not be fully secured, leading to repeated desaturations down to SpO2 76%. End-tidal CO2 at this time was 25 to 30 mmHg. 2 mg of midazolam, 0.4 mg of glycopyrolate, 25 mg of ketamine, and 30 mg of rocuronium were given to facilitate further investigation of the emergently placed ETT, which was determined via a fiberoptic bronchoscope (FOB) through the ETT, to be repeatedly migrating into the right mainstem bronchus. Sevoflurane was administered through emergent ETT at this point so as to maintain an anesthetic, as blood pressures were being maintained with mean arterial pressures around 100 without the use of any vasopressors. Surgical and anesthesia teams decided to replace the existing ETT to facilitate surgical exploration of the neck wound and repair of the tracheal injury.

**Figure 1:**
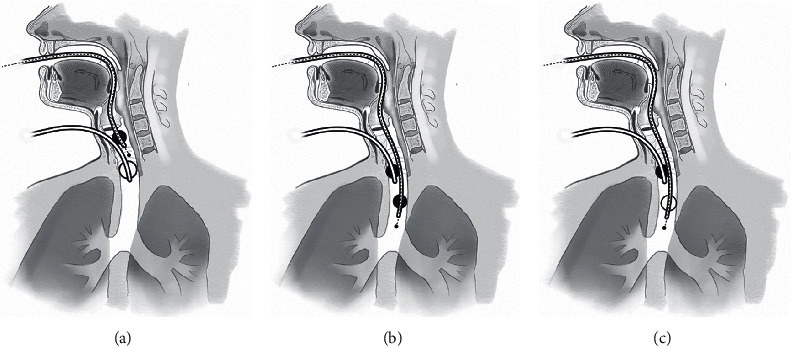
Simultaneous use of two endotracheal tubes. (a) The emergently placed ETT through the self-inflicted neck wound with the cuff inflated (white cuff) is used to ventilate the patient while a second ETT is passed from the oropharynx using a fiberoptic scope as a smart stylet (black dotted line in the tube). (b) The tracheal ETT cuff is deflated (black cuff) while the oral ETT is advanced over the fiberoptic scope. (c) The oral ETT cuff is inflated (white cuff) while the tracheal ETT is withdrawn and eventually taken out through the wound in the neck. A white cuff indicates an inflated cuff; a black cuff indicates a deflated cuff.

To accomplish this, the VL was placed through the patient's mouth and used to evaluate the posterior pharynx and glottic area which appeared normal ruling out cephalad extension of the injury. The lack of upper airway injury allowed for a clear view with the VL without obstruction with blood and secretions. With the VL in place, a FOB with a preloaded standard PVC 8.0 ETT was passed through the patient's mouth and through the vocal cords allowing visualization of the upper trachea which also appeared normal. Next, the surgical team manipulated the ETT through the trachea and tracheal wound such that the FOB was passed under the existing ETT. This maneuver allowed visualization of the posterior trachea at the level of the injury as well as the distal trachea both of which appeared normal. Then, the 8.0 ETT preloaded on the FOB was passed over the FOB beyond the level of the tracheal injury while leaving the first ETT in place (Figure 1). A size 8.0 ETT was chosen due to the possibility of prolonged postoperative ventilation and to allow for further airway interventions with bronchoscopy. Adequate chest rise and presence of end-tidal CO2 was confirmed with the second oral ETT before removal of the first tracheal ETT.

**Figure 2:**
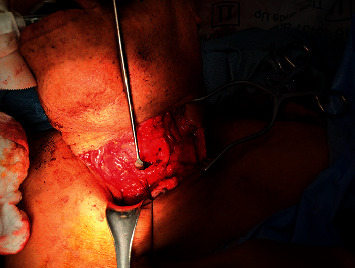
Tracheal injury.

Subsequent surgical exploration revealed that all structures adjacent to the tracheal injury including vascular structures were intact (Figure 2). A completion tracheostomy was then performed at the site of the tracheal injury. The ventilator was connected to the tracheostomy tube following confirmation of appropriate placement, and the patient was transferred to the ICU for further management. The patient had an uncomplicated course in the hospital wherein he was weaned off the ventilator the next day and his tracheostomy was decannulated shortly thereafter. He was transferred to inpatient psychiatry on postoperative day five and eventually discharged home on postoperative day twelve.

A photograph from the surgical field demonstrating the entry point into the trachea, taken after the second oral ETT was placed and the initial tracheal ETT was removed.

## 3. Discussion and Conclusions

We found this case to be unique and educational for a number of reasons including the simultaneous use of VL and FOB to assess the extent of the traumatic airway injury and the concurrent use of two 8.0 ETTs to ensure safe transition of the airway.

The simultaneous use of VL and FOB has been described in the literature in contexts ranging from the use as a teaching method, where an instructor uses a VL to monitor a trainee's use of an FOB [1], and to rescue in difficult airways [2–7]. With difficult airways, the FOB is often used as a “smart stylet” [8] to introduce an ETT directly into the trachea while the VL is used to visualize the glottic opening. One randomized controlled clinical trial was able to determine the superiority of a combined VL/FOB technique in successful intubation on first attempt, quicker intubation times, and less injury during intubation [9]. The utility of this combined approach in trauma patients with facial fractures has also been described [10]. However, to our knowledge, this is the first description of the use of VL and FOB in the assessment of a traumatic airway injury.

The ability to pass two 8.0 ETTs into the distal trachea was a surprising though welcome discovery. The average tracheal diameter for adult males is about 18–20 mm by radiographic imaging [11, 12]. Two 8.0 ETTs have a total outer diameter of 22 mm when placed side by side. We suspect that the injury to the trachea itself afforded some additional compliance permitting two simultaneous 8.0 ETTs to easily be placed. The intentional use of two single-lumen ETTs concurrently has been previously documented for purposes of single and differential lung ventilation [13–15]. However, this is the first description of side-by-side ETTs being used during a trauma and as a method to transition from a tracheal ETT to an oral ETT. Leaving the initial tracheal ETT in situ while placing the second, oral ETT allowed us to ensure that an adequate airway was always maintained. This is especially important in a traumatic airway as disruption of normal anatomy, and ongoing bleeding can make rescuing a lost airway difficult. To be successful, this maneuver required constant and clear communication between the anesthesia and surgical teams especially when the new ETT was being manipulated past the existing ETT at the level of the tracheal injury.

While there is no universal best method for dealing with penetrating neck injuries or traumatic airways in general, we believe this case demonstrates the utility of both FOB and VL used in conjunction to negotiate altered anatomy and, more interestingly, the possibility of two simultaneous ETTs *in vivo* so as to forgo the risk of losing an airway while securing a more reliable one.

## Abbreviations

ETT: Endotracheal tube
VL: Video laryngoscopy
FOB: Fiberoptic bronchoscopy
